# Predictive Value of Thyroglobulin Changes for the Curative Effect of Radioiodine Therapy in Patients With Metastatic Differentiated Thyroid Carcinoma

**DOI:** 10.3389/fendo.2021.667544

**Published:** 2021-05-10

**Authors:** Congcong Wang, Ruiguo Zhang, Renfei Wang, Zhaowei Meng, Guizhi Zhang, Feng Dong, Yajing He, Jian Tan

**Affiliations:** Department of Nuclear Medicine, Tianjin Medical University General Hospital, Tianjin, China

**Keywords:** differentiated thyroid carcinoma, radioiodine therapy, iodine radioisotope, curative effect, metastatic lesion, thyroglobulin change

## Abstract

**Background:**

Serum thyroglobulin (Tg) serves as a sensitive and easily available tumor marker for patients with metastatic differentiated thyroid carcinoma (m-DTC). The aim of the present study was to evaluate the predictive value of suppressed Tg changes (Δsup-Tg) and/or stimulated Tg changes (Δsti-Tg) to evaluate the efficacy of radioiodine therapy (RT).

**Methods:**

We studied 117 patients with m-DTC who received RT. Δsup-Tg and Δsti-Tg were compared after the first RT in different therapeutic response groups and a receiver-operating characteristic (ROC) curve was used to determine the cut-off values to predict non-remission. Univariate and multivariate analyses were used to investigate the effects of 17 observed factors on the efficacy of RT.

**Results:**

A total of 218 RT events in 117 patients with m-DTC were analyzed. After the last RT, the remission rate was 70.94% (83/117), and the proportion of remission events accounted for 74.77% (163/218). ROC curve analysis showed that the cut-off values for Δsup-Tg and Δsti-Tg after the first RT to predict the non-remission of RT were 21.54% and 27.63%, respectively. Age, the size of the metastasis, the maximum count of target metastatic lesions and the average count of contralateral non-target tissue on tomographic imaging (T_max_/NT_mean_) of the first RT, and Δsup-Tg after the first RT were identified as independent factors associated with RT efficacy.

**Conclusions:**

Tg response was valuable to predict RT efficacy for patients with m-DTC. Age, the size of the metastasis, T_max_/NT_mean,_ and Δsup-Tg after the first RT were verified as independent predictive factors of RT efficacy.

## Introduction

Differentiated thyroid carcinoma (DTC) accounts for approximately 90% of all thyroid carcinomas, and has a relatively good prognosis; however, 5-25% of patients with DTC develop distant metastasis (1–5). The 10-year survival rate of patients with metastatic differentiated thyroid carcinoma (m-DTC) is significantly reduced compared with patients with non-metastatic DTC (6). Radioiodine therapy (RT) has been recognized as a conventional therapeutic strategy for patients with ^131^I-avid m-DTC for nearly 80 years (6, 7). Studies have confirmed that RT can significantly reduce the tumor-related mortality of patients with m-DTC and improve their overall survival significantly (8, 9).

The 2015 American Thyroid Association (ATA) management guidelines pointed out that ^131^I-avid metastatic lesions can be treated with ^131^I, and the response to the previous RT and real-time disease status should be combined to assess whether the patient should undergo repeated RT (3, 4). Unfortunately, the guidelines failed to provide detailed indications for repeated RT. Therefore, how to evaluate the efficacy of RT on patients with m-DTC and how to determine the decision and timing of repeated RT have caused a considerable controversy, becoming important issues in current clinical work (1, 3, 4, 10).

A series of studies confirmed that multiple interrelated factors might change the uptake of ^131^I by metastatic lesions positively or negatively, and may ultimately change the RT outcome (1, 4, 10–12). Thyroglobulin (Tg) is an important and easily measured tumor marker for DTC, which reflects the patient’s tumor burden and is influenced by the levels of thyroglobulin antibody (TgAb) and thyroid stimulating hormone (TSH) (3, 4, 13). Meanwhile, Tg also has value to predict the response or resistance to RT.

Therefore, this retrospective clinical study aimed to evaluate the curative effect of RT by analyzing the clinical data of 117 patients with ^131^I-avid m-DTC, aiming to explore the value of Tg change in predicting the efficacy of RT, to identify relevant factors that affect RT efficacy, and provide a basis for clinical decision-making.

## Materials and Methods

### Study Conduct

The study protocol was approved by the Medical Ethics Committee of Tianjin Medical University General Hospital. All the patients who participated in this study provided written informed consent form before the start of the research. The authors performed anonymous analysis on all clinical data used in this study and fully guaranteed the accuracy and completeness of the data and analysis.

### Study Populations

We screened retrospectively 1821 patients with DTC who had received a total thyroidectomy and RT at our hospital from January 2013 to June 2019 using our institutional radionuclide therapy information system. Among these 1821 patients, we identified 117 patients with ^131^I-avid m-DTC with no thyroid tissue residue, as demonstrated using post-therapeutic ^131^I whole-body scanning (Rx-WBS) (78 female, 39 male, sex ratio (F:M) 2:1; age range: 11-74 years). Data comprising the clinicopathological characteristics of the patients at the time of diagnosis with DTC, the time of diagnosis of metastasis, lesion location and size, the changes in suppressed thyroglobulin (sup-Tg) and stimulated thyroglobulin (sti-Tg) before and after each RT, and the efficacy evaluation, were collected.

### Inclusion and Exclusion Criteria for This Study

The inclusion criteria of this study were as follows: (1) The patients underwent total or near total thyroidectomy. (2) The histopathological type was diagnosed as DTC. (3) The patient had undergone radioiodine residual ablation and no residual thyroid tissue was found upon Rx-WBS. (4) Focal or diffuse ^131^I-avid metastatic lesions were observed on Rx-WBS after excluding the physiological ^131^I uptake and contamination, with or without positive findings on other diagnostic/functional imaging modalities (computed tomography, CT; magnetic resonance imaging, MRI; ultrasonography, whole body bone static imaging, WBI; and Rx-WBS). (5) Negative TgAb. (6) Follow-up for more than six months after the last RT. The exclusion criteria comprised any of the following: (1) The patient did not undergo standard RT and efficacy evaluation. (2) Periodical follow-up data were incomplete. (3) The patient suffered from other malignant diseases.

### RT Procedures

The preparation and protocol of RT for patients with m-DTC were carried out according to the recommendations of the 2015 ATA guidelines (4). All the subjects strictly withdrew from levothyroxine and followed a low-iodine diet for 2-4 weeks before RT (TSH>30mIU/L) (1). Oral ^131^I at a dose of 5.55-7.40 GBq was given for each round of RT, and the interval between rounds of RT varied from 6 to 12 months (4, 14). The patients underwent Rx-WBS and tomographic image fusion was performed using a single photon emission computed tomography/computed tomography instrument (SPECT/CT, GE Discovery NM/CT 670; GE healthcare, Chicago, IL, USA) 3-7 days after RT (15). The region-of-interest (ROI) technique was used to analyze the uptake of ^131^I of the metastatic lesion on tomographic imaging semi-quantitatively (16). Briefly, the regions of each target metastatic lesion (T) and contralateral non-target tissue (NT) were delineated separately, and the maximum T count (T_max_) and the average count of NT (NT_mean_) were recorded. Consequently, T_max_/NT_mean_ was used as an index to evaluate the ability of the metastatic lesion to uptake ^131^I.

### Tg Assessment Profile

In this study, we performed separate quantitative analysis of sup-Tg and sti-Tg during RT. Sup-Tg was measured 3-4 months before and 2-3 months after RT, which was denoted as sup-Tg_1_ (TSH < 0.1mIU/L) and sup-Tg_2_ (TSH < 0.1mIU/L), respectively. The sti-Tg and TSH (TSH > 30mIU/L) on the day or within 3 days before RT (sti-Tg_1_, TSH_1_) and at the next (sti-Tg_2_, TSH_2_) time of RT were measured and recorded. Δsup-Tg, the change in the value of sup-Tg, was defined as follows: [(sup-Tg_1_ - sup-Tg_2_)/sup-Tg_1_ ×100%]. To eliminate the influence of TSH, we defined the change in the value of sti-Tg as follows: Δsti-Tg= [(sti-Tg_1_/TSH_1_- sti-Tg_2_/TSH_2_)/(sti-Tg_1_/TSH_1_) ×100%].

### Evaluation for the Efficacy of RT

According to the relevant literature (4, 6, 10, 11, 17), we combined the changes in serology and diagnostic imaging (Rx-WBS and ultrasound, CT, MRI, and WBI) to divide the efficacy of RT into a complete response (CR), partial response (PR), stable disease (SD), and progressive disease (PD). Then, patients with CR and PR were included in the remission group, while those with SD and PD were included in the non-remission group.

1. CR: No abnormal ^131^I uptake and the disappearance of all detectable metastatic lesions upon Rx-WBS and/or other imaging modalities, sti-Tg *<* 1ng/mL and/or sup-Tg *<* 0.2ng/mL.
2. PR: Decreased ^131^I uptake or reduced numbers of metastatic lesions upon Rx-WBS and/or other imaging modalities, with decreased sti-Tg and/or sup-Tg levels.
3. SD: No obvious change in the size of the metastatic lesions or ^131^I uptake upon Rx-WBS and/or other imaging modalities, with any change in sti-Tg and/or sup-Tg.
4. PD: Increased size or number of metastases, or higher ^131^I uptake upon Rx-WBS and/or other imaging modalities, with increased sti-Tg and/or sup-Tg levels.

### Statistical Analysis

IBM SPSS 26.0 software (IBM Corp, Armonk, NY, USA) was used to perform the statistical analysis of the data. Data are presented as means ± standard deviations, medians with ranges, numbers with percentages, or proportions. The Mann-Whitney U test was used to compare the Δsup-Tg and Δsti-Tg values in the different efficacy groups. Receiver-operating characteristic (ROC) curves were established to predict non-remission of RT for patients with m-DTC. The Mann-Whitney U test, chi-squared tests, or two-sample t test were performed for univariate analyses according to needs, and significant factors were then included in the logistic regression analysis to identify the independent risk factors that affect the efficacy of RT. P < 0.05 was considered statistically significant.

## Results

### Therapeutic Efficacy of RT

The 117 patients with m-DTC included in this study were followed-up regularly after receiving RT. The rates of CR, PR, SD, and PD were 19.66% (23 cases), 51.28% (60 cases), 16.24% (19 cases) and 12.82% (15 cases), respectively. The remission group (CR+PR) comprised 83 cases (70.94%), while the non-remission group (SD+PD) included 34 cases (29.06%). The enrolled patients with m-DTC received RT for one to five times, and a total of 218 RT events were finally recorded. Among them, the events of CR, PR, SD, and PD accounted for 10.55% (23/218), 64.22% (140/218), 18.35% (40/218), and 6.88% (15/218), respectively. The proportions of remission events (CR+PR) and non-remission events (SD+PD) were 74.77% (163/218) and 25.23% (55/218), respectively.

### Comparison of the Δsup-Tg and Δsti-Tg After The First/Single RT in Different Therapeutic Outcomes

The median Δsup-Tg after the first RT in the patients in the remission group was 31.68%, while that in the non-remission group was only 12.02%. There was a statistical difference between the two groups (z = -6.801, P < 0.001). Meanwhile, the median Δsti-Tg after the first RT in the patients in the remission group was 39.72%, which was also significantly higher than the 21.63% in the non-remission group (z =-5.961, P < 0.001) (**Table 1**).

**Table 1:** Comparison of the Δsup-Tg and Δsti-Tg values after the first or single RT for different therapeutic outcomes.

Efficacy	the first RT(n=117)		Efficacy of the event	single RT event(n=218)	
	Δsup-Tg (%)	Δsti-Tg (%)		Δsup-Tg (%)	Δsti-Tg (%)
Remission group	31.68(25.31,37.82)	39.72(31.82,53.63)	Remission event	29.06(22.10,35.10)	34.24(26.41,42.41)
Non-remission group	12.02(2.69,18.11)	21.63(3.08,30.45)	Non-remission event	5.99(-6.98,11.96)	12.54(-29.63,21.93)
Z	-6.801	-5.961	z	-10.298	-9.005
P-value	*<*0.001	*<*0.001	P-value	*<*0.001	*<*0.001

RT, radioiodine therapy.

Out of 218 RT events, remission RT was obtained for 163 events, while 55 yielded non-remission events. In the remission events, the median Δsup-Tg and Δsti-Tg after a single RT event were 29.06% and 34.24%, respectively, while they were only 5.99% and 12.54% in the non-remission events, which were statistically significantly different between the two types of therapeutic efficacy events (z =-10.298 and -9.005, both P < 0.001) (**Table 1**).

### ROC Curve Analysis of Δsup-Tg and Δsti-Tg After the First/Single RT to Predict the Curative Effect

**Figure 1:**
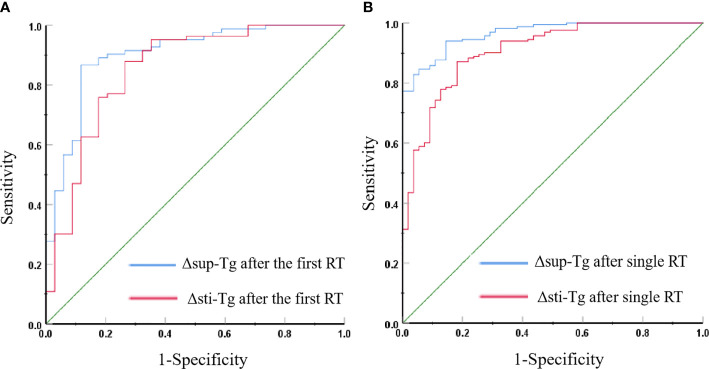
**(A)** Receiver operating characteristic (ROC) curves for Δsup-Tg and Δsti-Tg after the first RT to predict non-remission. **(B)** ROC curves for Δsup-Tg and Δsti-Tg after a single RT to predict non-remission.

The ROC curve showed that the Δsup-Tg and Δsti-Tg values after the first RT showed a good performance to predict non-remission (**Figure 1A**). A cut-off value of Δsup-Tg after the first RT at 21.54% was achieved to detect patients that did not achieve remission, with a sensitivity of 86.7% and specificity of 88.2%, and area under the curve (AUC) of 0.901. Meanwhile, a cut-off value of Δsti-Tg after the first RT at 27.63% was obtained, with a sensitivity of 88.0%, a specificity of 73.5%, and the corresponding AUC of 0.852, respectively. Thus, if the Δsup-Tg was lower than 21.54% and/or Δsti-Tg was lower than 27.63% after the first RT, the curative effect was more likely to be non-remission.

The cut-off value of Δsup-Tg after a single RT at 12.90% and Δsti-Tg after single RT at 22.69% were obtained by ROC curve analyses to best distinguish remission and non-remission events, with corresponding specificities of Δsup-Tg and Δsti-Tg after single RT separately of 85.5% and 81.8%, and sensitivities of 93.9% and 87.1%, and AUCs of 0.965 and 0.906, respectively (**Figure 1B**). That is, after single RT, a Δsup-Tg was less than 12.90%, and/or Δsti-Tg of less than 22.69% indicated that the single RT has poor efficacy.

### Univariate Analyses of the Efficacy of RT for m-DTC Patients

Upon analyzing the relationship between clinicopathological features and the therapeutic efficacy of ^131^I, a total of 17 factors were involved in the univariate analysis (**Tables 2**, **3**). We found that older patients, subjects with a bigger primary tumor, higher T stage, extrathyroidal invasion, BRAF^V600E^ mutation positive, metastatic lesions > 1cm, extrapulmonary distant metastases, higher sti-Tg levels at diagnosis, lower T_max_/NT_mean_ after the first RT, and those with lower Δsup-Tg and Δsti-Tg after the first RT had a higher probability of non-remission (P= 0.000, 0.000, 0.017, 0.027, 0.006, 0.000, 0.022, 0.003, 0.000, 0.000, and 0.000, respectively). However, we found no statistically significant differences in sex (P = 0.249), histological type (P = 0.208), N stage (P = 0.283), primary tumor multifocal (P = 0.587), diagnosis time of metastases (P = 0.105), and the first RT dose (P = 0.306).

**Table 2:** Univariate analyses for the continuous variables.

Characteristics	Remission group	Non-remission group	t or z	P-value
Age (year)	40.83 ± 12.61	55.03 ± 9.562	-5.902	<0.001
Primary tumor size (cm)	1.9(1.2,2.7)	2.8(1.9,3.6)	-3.501	<0.001
sti-Tg levels at diagnosis (μg/L)	63.60(15.32,159.00)	146.50(67.53,275.48)	-2.952	0.003
The first RT dose (GBq)	5.72 ± 1.25	5.97 ± 1.15	-1.028	0.306
T_max_/NT_mean_ of the first RT^a^	17.50(9.58,28.51)	8.14(4.63,14.26)	-4.824	<0.001
Δsup-Tg after the first RT (%)	31.68(25.31,37.82)	12.02(2.69,18.11)	-6.801	<0.001
Δsti-Tg after the first RT (%)	39.72(31.82,53.63)	21.63(3.08,30.45)	-5.961	<0.001

a The median T_max_/NT_mean_ is included in the study when the patient has multiple metastatic lesions. RT, radioiodine therapy.

**Table 3:** Univariate analyses for the categorical variables.

Characteristics	n	Remission group (%)	Non-remission group (%)	χ^2^	P-value
Sex				1.327	0.249
Male	39	25(64.10)	14(35.90)		
Female	78	58(74.36)	20(25.64)		
Histological type				1.584	0.208
Papillary	110	80(72.73)	30(27.27)		
Follicular	7	3(42.86)	4(57.14)		
T stage				10.146	0.017
T1	43	35(81.40)	8(18.60)		
T2	21	18(85.71)	3(14.29)		
T3	23	14(60.87)	9(39.13)		
T4	30	16(53.33)	14(46.67)		
N stage				2.525	0.283
N0	25	19(76.00)	6(24.00)		
N1a	18	10(55.56)	8(44.44)		
N1b	74	54(72.97)	20(27.03)		
Primary tumor multifocal				0.294	0.587
Yes	89	62(69.66)	27(30.34)		
No	28	21(75.00)	7(25.00)		
Extrathyroidal invasion				4.882	0.027
Yes	64	40(62.50)	24(37.50)		
No	53	43(81.13)	10(18.87)		
BRAF^V600E^ mutation				7.526	0.006
Positive	74	46(62.16)	28(37.84)		
Negative	43	37(86.05)	6(13.95)		
Diagnosis time of metastases				2.630	0.105
After RT	25	21(84.00)	4(16.00)		
Before RT	92	62(67.39)	30(32.61)		
The diameter of metastatic lesions				26.358	0.000
≤1cm	73	64(87.67)	9(12.33)		
>1cm	44	19(43.18)	25(56.82)		
Metastases sites				7.627	0.022
Only lung metastases	64	52(81.25)	12(18.75)		
Only lymphatic metastasis	24	15(62.50)	9(37.50)		
Multi-site metastasis	29	16(55.17)	13(44.83)		

T, tumor; N, lymph node. RT, radioiodine therapy.

### Multivariate Analyses of the Efficacy of RT for Patients With m-DTC

Multivariate logistic regression analysis was performed on efficacy-related factors from the univariate analysis. Multivariate logistic regression (**Table 4**) revealed that only the age, the size of the metastases, the T_max_/NT_mean_ of the first RT, and the Δsup-Tg after the first RT were verified as independent factors that predicted non-remission after RT. In summary, older patients with m-DTC (odds ratio [OR]: 1.180; 95% confidence interval [CI]: 1.063-1.308; P = 0.002), subjects with larger metastases (OR: 31.890, 95%CI: 2.832-359.162; P = 0.005), lower T_max_/NT_mean_ of the first RT (OR: 0.719, 95%CI: 0.568-0.910; P = 0.006), and lower Δsup-Tg after the first RT (OR: 0.832, 95%CI: 0.746-0.927; P = 0.001) had a higher probability of non-remission.

**Table 4:** Logistic regression analyses of the variables affecting non-remission after RT in 117 patients with m-DTC.

Characteristics	β	Standard error	Wald	*P* -value	*OR*	*95%CI*
Age	0.165	0.053	9.733	0.002	1.180	1.063-1.308
The diameter of metastatic lesions	3.462	1.235	7.853	0.005	31.890	2.832-359.162
T_max_/NT_mean_ of the first RT^a^	-0.330	0.120	7.539	0.006	0.719	0.568-0.910
Δsup-Tg after the first RT (%)	-0.184	0.055	11.070	0.001	0.832	0.746-0.927
Constant	-6.202	2.462	6.346	0.012	0.002	

a The median T_max_/NT_mean_ is included in the study when the patient has multiple metastatic lesions; β, regression coefficient; OR, odds ratio; CI, confidence interval; RT, radioiodine therapy.

## Discussion

Lungs, bones, and lymph nodes are the most common sites of DTC metastasis. The existence of metastatic lesions is the main cause of the decline in the quality of life and death of patients with m-DTC (6, 11, 18). If an early diagnosis and effective treatment are not undertaken, the 5-year mortality rate can be as high as 50% (19). ^131^I is one of the main methods of postoperative adjuvant treatment for patients with intermediate or high-risk DTC, which can significantly improve the overall survival rate and reduce the risk of recurrence, metastasis, and death (17, 20).

However, the conventional response evaluation criteria in solid tumors (RECIST) are usually imperfect to assess the RT response of patients with m-DTC, because un-measurable lesions are often found. In addition, because of the indolence and well differentiation of ^131^I-avid m-DTC, it is difficult to detect the morphological changes of the lesions in the early stage upon anatomical imaging (1, 6, 21). Although the response to therapy classification modified by the 2015 ATA guidelines can dynamically assess the patient’s disease status in real time (4), the effectiveness of RT cannot be adequately reflected because patients with ^131^I-avid m-DTC have been in a state of “structural incomplete response” under imaging evaluation for a long time.

Tg, an important biochemical marker for DTC, reflects the tumor burden of patients with DTC with total thyroidectomy accurately (4). The presence of TgAb will interfere with the determination of Tg and affect the accuracy of disease monitoring; therefore, the levels of Tg and TgAb were monitored simultaneously in this study and those subjects with TgAb positivity were excluded. Regular monitoring of Tg can be used to assist the evaluation of the efficacy of RT for patients with ^131^I-avid m-DTC, and a decrease in Tg is one of the important signs of effective treatment (13, 22, 23).

Therefore, the combined use of serological and imaging indicators has been established an indispensable tool to evaluate the efficacy of RT. Our previous study (17) showed that the sti-Tg value at diagnosis was an independent and significant predictor of RT efficacy in patients with DTC with pulmonary metastasis. The ROC curve analysis took a sti-Tg value of 55.50 ng/mL as the cut-off value, and the sensitivity and specificity of predicting non-remission was 78.7% and 73.1%, respectively. However, this was a static cut-off method to predict efficacy and judge prognosis, which did not consider the influence of the magnitude of ΔTg on prognosis during the treatment. Miyauchi et al. (24) proposed that the Tg doubling time could predict the recurrence and overall survival of patients with DTC. Barres et al. (25) reported that in patients with DTC treated using repeated RT, the reduction in sti-Tg after the first RT was related to prognosis. Patients with a 60% decrease in sti-Tg after the first RT were more likely to achieve CR upon subsequent RT. In this retrospective study, we found that patients with a decrease in sup-Tg of less than 21.54% and/or a decrease in sti-Tg of less than 27.63% after the first RT would have a higher probability of non-remission.

The uptake of ^131^I determines the dose of radiation absorbed by metastatic lesions, which directly affects the efficacy of RT (12). A prospective study of 77 patients with ^131^I-avid m-DTC in China showed that the maximum target/background ratio (T/B_max_) upon Rx-WBS and the change in the value of the sup-Tg level (ΔTg_on_%) after previous RT were associated independently with the outcome of the next RT (1). In the present study, for patients with m-DTC with multiple ^131^I-avid metastatic lesions, we took the median T_max_/NT_mean_ at the first RT as an index to evaluate the ^131^I uptake ability of the lesions. We found that the median T_max_/NT_mean_ of the first RT in the remission group (17.50) was significantly higher than that of the non-remission group (8.14).

The efficacy of RT toward m-DTC is affected by many factors. Studies have shown that in older patients, DTC accompanied by distant metastasis (lung and bone metastasis), large tumor size, and high sti-Tg before the first RT have poor therapeutic effects (10, 11, 17). The univariate and multivariate analysis in the present study showed that age, the size of the metastases, the T_max_/NT_mean_ of the first RT, and the Δsup-Tg after the first RT were related independently to the efficacy of RT. Notably, in predicting non-remission, the sensitivity and specificity of the Δsup-Tg value after the first RT at a cut-off value of 21.54% were 86.7% and 88.2%, respectively. This means that a lower decrease in sup-Tg after the first RT is directly associated with non-remission. The reason might be that older patients have a relatively long disease course and poor sensitivity to radiation. The poor uptake of ^131^I by the lesions would result in an insufficient absorbed dose of radiation in the lesions, thus affecting the efficacy of RT (2, 12). Therefore, the results allowed us to hypothesize that young patients with m-DTC with small metastatic lesions, good iodine uptake ability, and a Δsup-Tg after the first RT greater than 21.54% could benefit from repeated RT.

In this study, patients with extrathyroidal invasion had no abnormal neck uptake after successful radioiodine remnant ablation. The reasons may be as follows: Extrathyroidal invasion was discovered in the patients during surgery. After a standardized total or near total thyroidectomy, the tumor tissue was basically removed. After the patient was treated with radioiodine remnant ablation, the hidden tumor tissue was completely eliminated.

In terms of efficacy, patients with radioiodine refractory lesions (both with and without ^131^I-avid lesions) were classified into the non-remission group. For patients judged to have radioiodine refractory differentiated thyroid carcinoma (RAIR-DTC), especially those who did not absorb iodine or whose disease progressed despite absorbing iodine, RT termination may be considered. We should formulate an appropriate individualized follow-up treatment plan based on the patient’s condition (4). For some patients with RAIR-DTC with stable or slow progress, inoperable resection, and low tumor burden, the strategy of follow-up monitoring under TSH suppression therapy (TSH should be less than 0.1 mU/L) could be adopted. Local treatments (including surgical resection, external irradiation, and ablation) could be adopted for RAIR-DTC lesions with single occurrence, local clinical symptoms, and invasion of surrounding important organs and tissue structures. If the disease progresses rapidly, therapeutic strategies, such as targeted therapy, might be considered.

In RT decision-making and follow-up management for recurrent or metastatic DTC, sup-Tg and sti-Tg are important reference indicators to evaluate the response to RT, and a decrease in Tg often indicates a response to previous RT (4, 26). Our study further confirmed that not only can the trend of Tg be used to evaluate efficacy, but also the quantitative Tg change displayed a robust prediction of the outcome of RT. In brief, a decrease in sup-Tg less than 21.54% after the first RT indicated that patients had a higher probability of non-remission after RT.

Nevertheless, some limitations existed in this study. First, the presumed influence of the ^131^I remnant thyroid ablation on the effect of RT in the next course could not be determined definitively nor completely ruled out. Second, this retrospective single-center study did not include a large enough number of cases. Third, although we followed the inclusion criteria and exclusion criteria strictly to select the samples, selection bias might still exist because of the small number of cases eventually included in this study. Finally, the follow-up time was short, and thus the evaluation of long-term clinical outcome was lacking. Therefore, for the scalability of this study, we should seek multi-center institutions to establish a cut-off value of ΔTg to predict the efficacy of RT.

## Conclusion

In summary, the present study demonstrated that most patients with m-DTC could benefit from RT. Older subjects with metastases *>* 1 cm, lower T_max_/NT_mean_ and lower Δsup-Tg after the first RT were less likely to experience remission. The optimal cut-off value for Δsup-Tg after the first RT to predict RT efficacy for m-DTC was 21.54%. These findings have important guiding significance for the optimization of treatment strategies and the evaluation of prognosis of patients with m-DTC.

## Data Availability Statement

The datasets used during the present study are available from the corresponding author upon reasonable request.

## Ethics Statement

The studies involving human participants were reviewed and approved by Ethical Committee of Tianjin Medical University General Hospital. Written informed consent to participate in this study was provided by the participants’ legal guardian/next of kin.

## Author Contributions

CW and RW contributed to the conception and design of the study. CW, RZ, and FD assisted with data acquisition. CW, RZ, and RW conducted the statistical analyses and drafted the manuscript. JT, ZM, YH, GZ, and FD critically revised the manuscript. All authors contributed to the article and approved the submitted version.

## Funding

This study was sponsored by the National Natural Science Foundation of China (Grant No. *81801732* and *81501510*).

## Conflict of Interest

The authors declare that the research was conducted in the absence of any commercial or financial relationships that could be construed as a potential conflict of interest.
